# Cerebral hemodynamic abnormalities of patients with ischemic stroke who are opium addicted: A study by transcranial doppler ultrasonography

**Published:** 2019-04-04

**Authors:** Yaser Moadabi, Alia Saberi, Sajjad Hoseini, Ashkan Karimi, Shahrokh Yousefzadeh-Chabok

**Affiliations:** 1 Neuroscience Research Center, Guilan University of Medical Sciences, Rasht, Iran; 2 Department of Neurology, Poursina Hospital, Guilan University of Medical Sciences, Rasht, Iran

**Keywords:** Opium, Doppler Transcranial Ultrasonography, Stroke, Blood Flow Velocity

## Abstract

**Background:** Ischemic stroke as the major cause of mortality and morbidity worldwide has different risk factors. One of its modifiable risk factors is opium addiction whose role is not clear yet. This study aimed at assessing the hemodynamic parameters in ischemic stroke patients addicted to opium using transcranial Doppler (TCD) ultrasonography and comparing them with non-addicted patients.

**Methods:** This comparative cross-sectional study was conducted in an academic hospital in the north of Iran in 2016. All the patients diagnosed as ischemic stroke underwent cerebrovascular ultrasound in the first 4 days of symptoms onset. Frequency of hemodynamic abnormalities confirmed by pulsatility index (PI) and mean flow velocity (MFV) were determined and compared between the two groups. The data were analyzed by chi-square test, t-test, and multiple logistic regression models using SPSS software.

**Results:** A total of 353 patients with ischemic stroke (92 addicted and 261 non-addicted patients) were enrolled in the study. Univariate analysis indicated that the PI and MFV differences were statistically significant between two groups of addicted and non-addicted patients (P = 0.0001). By multivariate logistic regression model, the age [odds ratio (OR) = 1.143], diabetes mellitus (DM) (OR = 3.875), hypertension (HTN) (OR = 2.557), and opium usage (OR = 9.615) had influence on PI and only opium usage (OR = 3.246) had influence on MFV.

**Conclusion:** Opium usage affects the cerebral hemodynamic parameters and increases the chance of having abnormal PI as ten-fold and abnormal MFV as three-fold.

## Introduction

Cerebral stroke is a leading cause of death and disability in the world and also in Iran, causing high mortality rate and wasting considerable money in the treatment.^   1 ^ A large number of people suffering from this disease are in an adverse condition both socially and financially.^   2 ^ The total financial burden of stroke in 2006 was 9.57 billion dollars, and for each patient, it was more than 140048 dollars.^   3 ^

As stroke is mostly preventable, conducting further research in this field and obtaining epidemiological data in association with stroke and its risk factors in our country, Iran, can diminish its incidence and related costs. The risk factors associated with ischemic stroke can be categorized as preventive and non-preventive. Non-preventive risk factors are age, male gender, race, family history, a former episode of stroke, and genetic, hormonal, and environmental factors. Preventive factors are smoking, low socioeconomic status (SES), hypertension (HTN), dyslipidemia, hyperuricemia, cardiovascular disorders, carotid artery diseases, and obesity.^4^^-^^7^ Some of the abovementioned risk factors have role on prognosis and also hospitalization period which are responsible for a part of cost and economic burden of stroke.^8^

The major drugs abused in Iran are opium and its byproducts.^9^ Considering the probable role of opium in stroke and the shortage of research in this field, we aimed to study the hemodynamic indexes of brain blood vessels with transcranial Doppler (TCD) sonography in opium and non-opium subcategories in patients with ischemic stroke. TCD is a noninvasive examination method utilizing the good penetrability of low-frequency ultrasound and low-frequency ultrasonic probe to detect the blood flow velocity and hemodynamics of intracranial blood vessels.^10^ In this regard, it is expected to find the hypothetical pathophysiology underlying the effect of opium on brain blood vessels.

## Materials and Methods

This comparative cross-sectional study was conducted in an academic hospital in the north of Iran on patients with stroke which was confirmed with brain computed tomography (CT) scan and neurologic assay in 2016. It was executed after being confirmed by Ethics Committee of Guilan University of Medical Sciences, Rasht, Iran (ethic code: IR.GUMS.REC.1395.43). 

Inclusion criteria were ischemic stroke and age over than 40 years. Exclusion criteria were hemorrhagic stroke, using ventilator and supportive care as the obstacle to perform TCD, and having inappropriate view in TCD. The patients who had the history of opium usage before 1 year ago and now were not using it, were also excluded from the study. Moreover, having hemoglobin (Hb) level lower than 10 g/dl and partial pressure of carbon dioxide (PCo_2_) more than 40 mmHg were considered as exclusion criteria.

To calculate the sample size in this study, the data of Hamzei Moqaddam, et al. study^9^ were used. Based on the following formula, the minimum sample size required in this study was 140 persons for each group.

**Figure:**
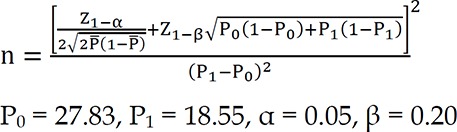


Owing to the limited number of stroke patients with a history of addiction, the following correction formula was used for sample size in stroke groups with and without history of addiction. For this purpose, the ratio of 3 to 1 was used.

**Figure:**
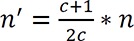


Considering that the sample size was 140 patients and the ratio of 3 (control group) to 1 (case group) was used, the sample size needed in this study included 90 opium-dependent patients for the case group and 270 non-dependent patients for the control group.

Opium dependency was defined based on Diagnostic and Statistical Manual of Mental Disorders-4^th^ Edition (DSM-IV) criteria including dysphoric mood, nausea or vomiting, muscle aches, lacrimation or rhinorrhea, pupillary dilation, piloerection, sweating, diarrhea, yawning, fever, and insomnia. Duration of use was considered one year. 3 out of 9 items should be met to call a patient an opium-dependent person. The patients were enrolled in this study after fulfilling an informed consent (by patients or their legal relatives).

In 4 days, after the onset of stroke, a neurologist performed the TCD (device from Atys Company, France) for each patient. Middle cerebral artery (MCA) was assessed from temporal window. 

**Table 1:** Demographic and transcranial Doppler (TCD) variables of studied patients including opium users and  non-opium users

**Variable**		**Opium users**	**Non-opium users**	**Total patients**	**P**
Sex [n (%)]	Male	70 (76.1)	127 (48.7)	197	0.0001
	Female	22 (23.9)	134 (51.3)	156	
Abnormal PI [n (%)]		64 (69.6)	69 (26.4)	133	0.0001
Abnormal MFV [n (%)]		21 (22.8)	17 (6.5)	38	0.0001
DM [n (%)]		24 (26.1)	81 (31.0)	105	0.4270
HTN [n (%)]		25 (27.2)	106 (40.0)	131	0.0240
Smoking [n (%)]	Yes	36 (39.1)	22 (8.4)	58	0.0001
	No	47 (51.1)	235 (90.0)	282	
	Quit	9 (9.8)	4 (1.5)	13	
HLP [n (%)]		3 (3.3)	4 (1.5)	7	0.3830
Age (year) (mean ± SD)		69.55 ± 10.99	66.58 ± 11.29	67.35 ± 11.27	0.0300

PI: Pulsatility index; MFV: Mean flow velocity; DM: Diabetes mellitus; HTN: Hypertension; HLP: Hyperkeratosis lenticularis perstans; SD: Standard deviation

In TCD, a wave, which cannot be heard by the human ear is transferred through the skull tissue and provides information such as pulsatility index (PI), maximum systolic velocity, and mean flow velocity (MFV). The PI more than 1.1 and MFV more than 120 cm/s in MCA were considered abnormal. 

For data analysis in this study, we used independent t-test, chi-square test, and multivariate logistic regression model in SPSS software (version 21, IBM Corporation, Armonk, NY, USA). The level of significance was considered < 0.05.

## Results

In this study, 353 patients with the mean age of 67.35 ± 11.27 years were included. A total of 26.1% of patients (92 cases) were opium users. Table 1 explains the demographic information and TCD variables.

As table 1 shows, the differences of PI and MFV were statistically significant between the two groups (P = 0.0001). 

We assessed the effect of each background factor on PI and MFV by univariate analysis (Table 2). Age had effect on PI (P < 0.001), but not on MFV (P = 0.849). In addition, according to table 2, sex, opium usage, diabetes mellitus (DM), HTN, and smoking were significantly associated with effects on the patients’ PI. Furthermore, sex, opium usage, and smoking were significantly associated with the patients’ MFV.

Then, to eliminate the confounding effect of background factors on PI and MFV, and to find the pure effect of opium on the patients’ hemodynamic variables, the variables with P-value of less than 0.25 were entered in the logistics regression model using the enter method to assess the pure effect of opium dependency on PI and MFV (Table 3).

**Table 2:** Assessment of the effect of each background factor of patients on pulsatility index (PI) and mean flow velocity (MFV) by univariate analysis

**Variable**		**Total**	**Abnormal PI increase** **[n (%)]**	**P**	**Abnormal MFV increase** **[n (%)]**	**P** *
Sex	Male	197	86 (43.7)	0.0110	29 (43.7)	0.0090
	Female	156	47 (30.1)		9 (5.8)	
Opium usage	Yes	92	64 (69.6)	< 0.0001	21 (22.8)	0.0001
	No	261	69 (26.4)		17 (6.5)	
DM	Yes	105	49 (46.7)	0.0300	12 (11.4)	0.8510
	No	248	84 (33.9)		26 (10.5)	
Smoking	Yes	58	26 (44.8)	< 0.0001	13 (22.4)	0.0070
	No	282	95 (33.7)		24 (8.5)	
	Quit	13	12 (92.3)		1 (7.7)	
HTN	Yes	131	64 (48.9)	0.0010	14 (10.7)	> 0.9999
	No	222	69 (31.1)		24 (10.8)	
HLP	Yes	7	5 (71.4)	0.1090	0 (0)	> 0.9999
	No	346	128 (37.0)		38 (11.0)	

PI: Pulsatility index; MFV: Mean flow velocity; DM: Diabetes mellitus; HTN: Hypertension; HLP: Hyperkeratosis lenticularis perstans

* Fisher's exact test

**Table 3:** Logistics regression model for determination of effective factors on pulsatility index (PI) and mean flow velocity (MFV)

**Variable**		**B**	**SE**	**P**	**OR**	**95% CI for Exp (B)**	
						**Lower**	**Upper**
PI	Age	0.133	0.018	< 0.0010	1.143	1.103	1.184
	Sex	-0.591	0.315	0.0610	0.554	0.299	1.028
	Opium usage	-2.260	0.361	< 0.0010	0.104 (9.615)	0.051	0.212
	DM	-1.356	0.346	< 0.0010	0.258 (3.875)	0.131	0.508
	HTN	-0.938	0.303	0.0020	0.391 (2.557)	0.216	0.709
	Smoking	-0.047	0.355	0.8940	0.954	0.476	1.911
	HLP	-1.793	1.174	0.1270	0.167	0.017	1.664
	Constant	2.386	2.648	0.3680	10.866	-	-
MFV	Sex	-0.641	0.423	0.1300	0.527	0.230	1.208
	Opium usage	-1.178	0.375	0.0020	0.308 (3.246)	0.148	0.642
	Smoking	-0.461	0.353	0.1920	0.631	0.316	1.261
	Constant	1.489	0.791	0.0600	4.433	-	-

SE: Standard error; OR: Odds ratio; CI: Confidence interval; PI: Pulsatility index; DM: Diabetes mellitus; HTN: Hypertension; HLP: Hyperkeratosis lenticularis perstans; MFV: Mean flow velocity

As table 3 shows, age (OR = 1.143), DM (OR = 3.875), HTN (OR = 2.557), and opium usage (OR = 9.615) had influence on PI and only opium usage (OR = 3.246) had influence on MFV.

## Discussion

In this study, 353 patients were included and 26.1% of them (92 cases) were opium users. The results demonstrated that PI and MFV differences were statistically significant between the opium users and non-opium users. After eliminating confounding effects, it was concluded that age, DM, HTN, and opium usage had influence on PI, and only opium usage had influence on MFV. As opium usage increases, the chance of having abnormal PI increases to seven-fold and chance of having abnormal MFV to three-fold. 

The difference between the groups in terms of age was significant. The mean age in the group with dependency was 69.55 ± 10.99 years compared to non-users with the mean age of 66.58 ± 11.29, being inconsistent with the results of Hamzei Moqaddam, et al.^9^ survey, owing to different data collection methods. The peak age for stroke is over 60, and at this age, a majority of drug users tend to use opium compared to younger users preferring other drugs.

Tegeler, et al.^11^ showed that as age proceeds, the blood flow velocity in brain vessels decreases and PI increases. This decrement in men older than 40 years is more than women which is probably due to less hematocrit in female gender.^11^^,^^12^ In our study, with increasing age, the MFV did not change, but the chance of abnormal increasing of PI increased to 1.14 per one year of age. 

Zarei, et al. study shows that intracranial and extracranial vessels are narrow in patients with a history of HTN and DM.^13^ In Lee, et al.^14^ study, age, DM, and HTN are predictive factors of PI increase. These findings are compatible with our results in which age, DM, and HTN had influence on PI, but not on MFV. PI reflects the intracranial vessels resistance that its increasing is due to narrowing of the small vessels as a result of lipohyalinosis and atherosclerosis in patients with DM and HTN. 

According to Tegeler, et al.^11^ study, usage of cocaine and heroin can be associated with increase in PI, which can be reduced by methadone therapy. Nevertheless, our results demonstrated that the usage of opium increased the chance of increasing of PI and MFV. However, the mechanism of effect of cocaine on cerebral hemodynamic parameters is different from opium. It seems that the effect of methadone and traditional opium should be similar, but according to Tegeler, et al. study and our study, it seems that the effect of traditional opium is contrary to methadone. Unfortunately, the studies are few in this field.^11^

In terms of effect of opium usage on incidence of stroke, the results are contrary. 

The study conducted by Sloan, et al. showed that drug usage could increase the incidence of stroke six-fold and even if it was used at age under 35, it would increase the chance of stroke by 11-fold. However, the effects of different drugs vary, and the complications of using some drugs such as heroine, amphetamines, and cocaine are greater.^15^ Rezvani and Ghandehari study revealed a significant association between opium usage and ischemic stroke.^16^ Nevertheless, in one study by Ghayeghran, et al., no relationship was found between ischemic stroke and opium usage.^17^

Ghanei, et al., by assessing the effects of opium on vessels’ endothelium, showed an association between drug abuse and inflammation of vessel’s endothelium confirmed by increasing c-reactive protein (CRP) and decreasing nitric oxide (NO).^2^ Moreover, there are studies indicating a relation between opium dependency and ischemic heart diseases. In this respect, the results of the studies conducted by Hashemi, et al.^18^ and Rezaei, et al.^19^ indicate that opium abuse has aggravating effects on atherosclerosis of coronary arteries. Another study demonstrated that opium abuse could increase the risk of cardiovascular diseases (CVDs) by increasing plasma homocysteine.^20^ Patients with atherosclerosis have a higher level of interleukin-1 receptor antagonist (IL-1Ra), which is less in non-opium patients. This can show the negative effect of opium on the cardiovascular system.^21^ Hamzei Moqaddam, et al. study shows that opium abuse can increase plasma fibrinogen which can independently cause and progress atherosclerosis in coronary, peripheral, and brain vessels.^9^ Its effect on oxidative stress which play role in stroke^22^ is not yet established. It is predicted that opium can have the same damaging effect on brain vessels similar to coronary vessels.^23^

However, the influence mechanisms of opium on cerebral hemodynamic parameters should be assessed, which in some parts are different from the stroke mechanisms.

## Conclusion

Opium usage affects cerebral hemodynamic parameters including PI and MFV in patients with ischemic stroke, increasing the chance of having abnormal PI as seven-fold and abnormal MFV as three-fold. Considering that PI and MFV elevate in cerebral microvascular stenosis and large vessel stenosis, respectively, it can be concluded that opium impacts both small and large vessel stenosis and related strokes.

## Notes:

**How to cite this article:** Moadabi Y, Saberi A, Hoseini S, Karimi A, Yousefzadeh-Chabok Sh. Cerebral hemodynamic abnormalities of patients with ischemic stroke who are opium addicted: A study by transcranial doppler ultrasonography. Iran J Neurol 2019; 18(2): 76-81.
